# A Mixed Methods Study to Explore Relevant Metrics for a Results Framework Measuring the Public Health Impact of Reliance-Based Pathways

**DOI:** 10.1007/s43441-023-00559-5

**Published:** 2023-08-08

**Authors:** Inez Adams, Patricia A. Cuff, Lawrence Liberti

**Affiliations:** 1 Health and Medicine Division, National Academies of Sciences, Engineering, and Medicine, 500 Fifth St., NW, Washington, DC 20001 USA; 2 The D.K. Kim International Center For Regulatory Science, University of Southern California, Alfred E. Mann School of Pharmacy and Pharmaceutical Sciences, 1540 Alcazar Street, Los Angeles, CA 90089 USA

**Keywords:** Results framework, Regulatory reliance, Public health, Reliance-based pathways, Metrics

## Abstract

Reliance-based pathways for the marketing authorization of medical products have been identified as valuable regulatory tools for the timely provision of effective, safe, quality medicines for people worldwide; however, little research has been conducted on the best way to measure the public health impact of using reliance-based pathways. The current mixed methods study was designed to explore which characteristics or “metrics” could be used to measure the impact of reliance-based pathways. A quantitative survey (*n* = 70) and in-depth interviews (IDIs) (*n* = 10) were employed to query various stakeholders (e.g., industry, regulatory authorities, NGOs) about the metrics they believed would be important to include in a framework designed to measure the impact of reliance-based regulatory pathways on advancing public health. Based on survey results, (1) ability to meet targeted product assessment timeline, (2) increased access to expertise, which is limited or not available in the agency, (3) shortened median number of days (annually) to market for medical products, (4) lower morbidity and mortality rates due to greater access to medical products, and (5) movement toward technical standards harmonization were the Top Five most important metrics to be included in a framework. IDI results suggest that, while important, the relevance of the Top Five metrics may vary by region or regulatory authority. Interviewed stakeholders intuitively believe reliance-based regulatory pathways are a worthwhile endeavor; however, there must be “harmonization” within the reliance ecosystem that creates a strong understanding of the factors necessary for reliance-based pathways to be utilized in a successful manner.

**Supplementary Information:**

The online version contains supplementary material available at 10.1007/s43441-023-00559-5.

## Introduction

Reliance-based pathways for the marketing authorization of medical products are valuable regulatory tools for nations wishing to assure the timely provision of effective, safe, quality medicines for their people. The use of such pathways is enshrined in the World Health Organization’s *Good Reliance Practices* guideline [1]. Reliance-based pathways can span from informal work-sharing arrangements to using other trusted regulatory authorities’ (RA) work products (e.g., scientific assessment and/or inspection reports) to inform an agency’s own regulatory decisions, as in the case of abridged, abbreviated, or verification review processes [2]. They can also be more formalized as happens with mutual recognition agreements, in which the regulatory decision of a trusted reference agency is deemed valid for another jurisdiction (e.g., a verification review) [2]. Whether the regulatory authority takes into account the work of a trusted reference agency (i.e., reliance) or routinely accepts the regulatory decision of another (i.e., recognition), each nation retains sovereignty over and responsibility for its own decisions. While the use of reliance-based pathways during a public health crisis like the COVID-19 pandemic has been largely embraced, participation in reliance partnerships for non-emergency situations has often been met with resistance [2].

*Regulating Medicines in a Globalized World: The Need for Increased Reliance Among Regulators*, a U.S. Food and Drug Administration-commissioned National Academies of Sciences, Engineering, and Medicine study, examined the challenges to and opportunities for increasing reliance activities among regulatory authorities [2]. Protecting and promoting public health through the independent regulatory assessment of medicines marketing applications, the report stated, is a priority objective for all regulatory agencies; however, limited human and financial resources and the increasing complexity of medical products present challenges to meeting this objective. Reliance-based pathways can reportedly mitigate some of these challenges, as RAs are able to rely on work conducted by other trusted RAs to obtain needed scientific resources [3]. Mutual trust, open sharing of helpful information, and sovereignty of decision-making are principles that have been identified as being key to unilateral, bilateral, or multilateral reliance processes [4].

A challenge presented by the NASEM committee was how to measure the public health impact of using reliance-based pathways [2]. While positive impacts on public health seem intuitive with increased reliance, the committee noted a dearth of metrics for evaluating the public health impacts (positive or negative) of reliance-based agreements. According to Zall Kusek and Rist [5], using a results framework, could potentially broaden the foci from monitoring reliance agreements to measuring impact, which is consistent with the public health missions of most regulatory agencies. Separate from the report, several advantages to using a results framework were outlined by the USAID funded program, “Data for Impact” [6]. Embedding a results framework into an organization helps create a culture of critical self-examination to build a strong, more effective system where everyone at all levels of the organization is accountable for achieving its targeted results.

The current mixed methods study was designed to explore which characteristics or “metrics” could be used to measure the various impacts of reliance-based pathways. A quantitative survey and in-depth interviews (IDIs) were employed to query experts involved in the assessment of medical products about the metrics they believe would be important to include in a framework designed to measure the impact of reliance-based pathways on regulatory agencies and on advancing public health.

## Methods

This study was conducted independent of the aforementioned U.S. Food and Drug Administration-commissioned NASEM study, *Regulating Medicines in a Globalized World: The Need for Increased Reliance Among Regulators* [2]. The protocol for this study was reviewed by the National Academies of Sciences, Engineering, and Medicine’s Institutional Review Board. All participants volunteered to participate.

### Quantitative Survey

#### Survey Design

A series of literature searches and informal discussions with subject matter experts were carried out by the authors of this paper after the 2020 NASEM report [2] was released. Based on these inputs, a list of potential metrics to assess the impacts of reliance-based pathways were identified. These metrics were used to develop a 12-item online survey. The survey was prepared by the authors and sent to eight subject matter experts for pilot testing. After pilot testing, the survey was updated and 810 individuals expressing an interest in the NASEM report [2] as well as individuals from the authors’ professional networks (e.g., LinkedIn contacts) were invited to participate in the survey.

At the beginning of the survey, respondents were asked to identify their affiliation, the income level of the country or region in which they primarily worked, their participation in regulatory assessments, and the types of regulatory activities in which they participated. Participants were asked to rate the importance of 11 metrics—selected based on the literature and subject matter expert advice—in a framework that would measure the impacts of reliance-based pathways on a 5-point Likert scale, ranging from “not at all important” to “extremely important.” In addition to rating the pre-selected metrics, respondents were given the option to add and rate two additional self-selected metrics not on the original list. Respondents were then asked to choose and rank the Top Five metrics they thought should be included in the framework, selecting from the pre-selected metrics as well as the metric/s they provided. The final question of the survey was open-ended and provided an opportunity for respondents to offer any additional comments regarding potential reliance-based regulatory pathways metrics.

#### Data Analysis

Box 1 shows the calculations used to determine the rank order of the 11 metrics. The ranking score for each of the 11 metrics was calculated by multiplying the number of responses in each ranked position (i.e., position 1 through position 5) by the weight of each ranked position (e.g., position 1 is 5 times the weight of position 5).

**Box 1:** Calculations for Determining Rank Order of Metrics

(*a*1 × *b*1) + (*a*2 × *b*2) + (*a*3 × *b*3) + (*a*4 × *b*4) + (*a*5 × *b*5) = rank score	
*a* = number of responses in ranked position, *b* = weight of ranked position	
Position 1 has a weight of 5; position 2 has a weight of 4; position 3 has a weight of 3; position 4 has a weight of 2; and position 5 has a weight of 1	
a. Ability to meet targeted product assessment timeline	
(14 × 5) + (7 × 4) + (6 × 3) + (7 × 2) + (6 × 1) = 136	
b. Increased access to expertise, which is limited or not available in the agency	
(9 × 5) + (6 × 4) + (7 × 3) + (7 × 2) + (6 × 1) = 110	
c. Shortened median number of days (annually) to market for medical products	
(5 × 5) + (7 × 4) + (8 × 3) + (7 × 2) + (8 × 1) = 99	
d. Lower morbidity and mortality rates due to greater access to medical product	
(10 × 5) + (6 × 4) + (6 × 3) + (0 × 2) + (5 × 1) = 97	
e. Movement toward technical standards harmonization	
(5 × 5) + (3 × 4) + (9 × 3) + (10 × 2) + (10 × 1) = 94	
f. Increased access to more inspection data available when regulatory decisions are made	
(5 × 5) + (7 × 4) + (7 × 3) + (3 × 2) + (5 × 1) = 85	
g. Expansion of RA’s capacity to perform work (e.g., number of inspections, number of assessments)	
(4 × 5) + (7 × 4) + (8 × 3) + (5 × 2) + (3 × 1) = 85	
h. Increased number of reliance partnerships with which an RA is engaged	
(1 × 5) + (4 × 4) + (2 × 3) + (7 × 2) + (2 × 1) = 43	
i. Cost savings for consumers	
(1 × 5) + (4 × 4) + (3 × 3) + (5 × 2) + (2 × 1) = 42	
j. Increased number of marketing application reviews annually for the RA	
(3 × 5) + (2 × 4) + (1 × 3) + (5 × 2) + (6 × 1) = 42	
k. Cost savings for RA	
(2 × 5) + (1 × 4) + (3 × 3) + (1 × 2) + (5 × 1) = 30	

### Qualitative Individual In-Depth Interviews (IDIs)

#### Data Collection

To further explore the survey findings and inform the proposed framework, we invited survey respondents who agreed to be contacted to participate in 30-min IDIs. In addition, experts in the field of regulatory reliance within the authors’ networks were invited to participate. The authors of this paper interviewed a convenience sample of 10 participants with one author leading the discussions. The interviewers presented the participants with the Top Five metrics, as identified in the survey results, and asked them to share their thoughts about the metrics. Specifically, they were asked if they agreed with the Top Five metrics and if not, why. Participants were also asked to provide their insights about how, if at all, each metric was used in practice; whether the metric had nuances that should be considered; the feasibility of collecting data for that metric; and the relevance of the metric for the assessment of a reliance-based pathway. Additional follow-up questions were asked based on the discussions and whether the participants had used any of the metrics. Participants were also asked to provide alternative metrics that might replace any of the Top Five.

#### Data Analysis

Qualitative IDIs were conducted and recorded via Zoom video conferencing. IDIs were transcribed verbatim for data analysis. A codebook that focused on research objectives was developed. In qualitative research, a codebook is a framework that outlines how themes and patterns will be identified and captured for analysis. IDIs were manually coded. Two researchers analyzed transcripts for themes and patterns. Content analysis used both inductive and deductive coding in order to analyze IDIs for known research objectives and to detect patterns that emerged organically.

## Results

### Quantitative Survey

#### Sample

Seventy-six individuals responded to the survey; however, data from six respondents were removed due to missing data in key fields. The 70 individuals in the final sample represented the following organizations: pharmaceutical industry (*n* = 38), regulatory authority (RA) (*n* = 15), non-governmental organization (*n* = 6), academia (*n* = 4), government other than RA (*n* = 2), procurement agency (*n* = 1), other (*n* = 4).

#### Survey Results

Box 2 lists the 11 metrics provided in the quantitative survey (Top Five in priority order).

**Box 2:** Eleven Metrics Considered for the Framework

∙ Ability to meet targeted product assessment timeline (#1)
∙ Increased access to expertise, which is limited or not available in the agency (#2)
∙ Shortened median number of days (annually) to market for medical products (#3)
∙ Lower morbidity and mortality rates due to greater access to medical products (#4)
∙ Movement toward technical standards harmonization (#5)
∙ Increased access to more inspection data available when regulatory decisions are made
∙ Expansion of RA’s capacity to perform work (e.g., number of inspections, number of assessments)
∙ Increased number of reliance partnerships with which an RA is engaged
∙ Cost savings for consumers
∙ Increased number of marketing application reviews annually for the RA
∙ Cost savings for RA

Respondents were invited to provide additional metrics they thought would be relevant to include in a framework measuring the public health impact or efficacy of reliance-based pathways. In response, 56 entries were generated (See Supplementary Material “Other metrics” to be Included in a Framework to Measure the Public Health Impact of a Reliance Agreement (Open-ended responses)). Of the 56 entries, 51 were novel (i.e., different from the original 11). Participant responses that directly or indirectly reflected metrics from the original list of 11 (e.g., cost savings, ability to meet targeted product assessment timeline) were pulled out and are shown in the supplementary materials as “Responses that reiterated the original 11 metrics.”

**Figure 1:**
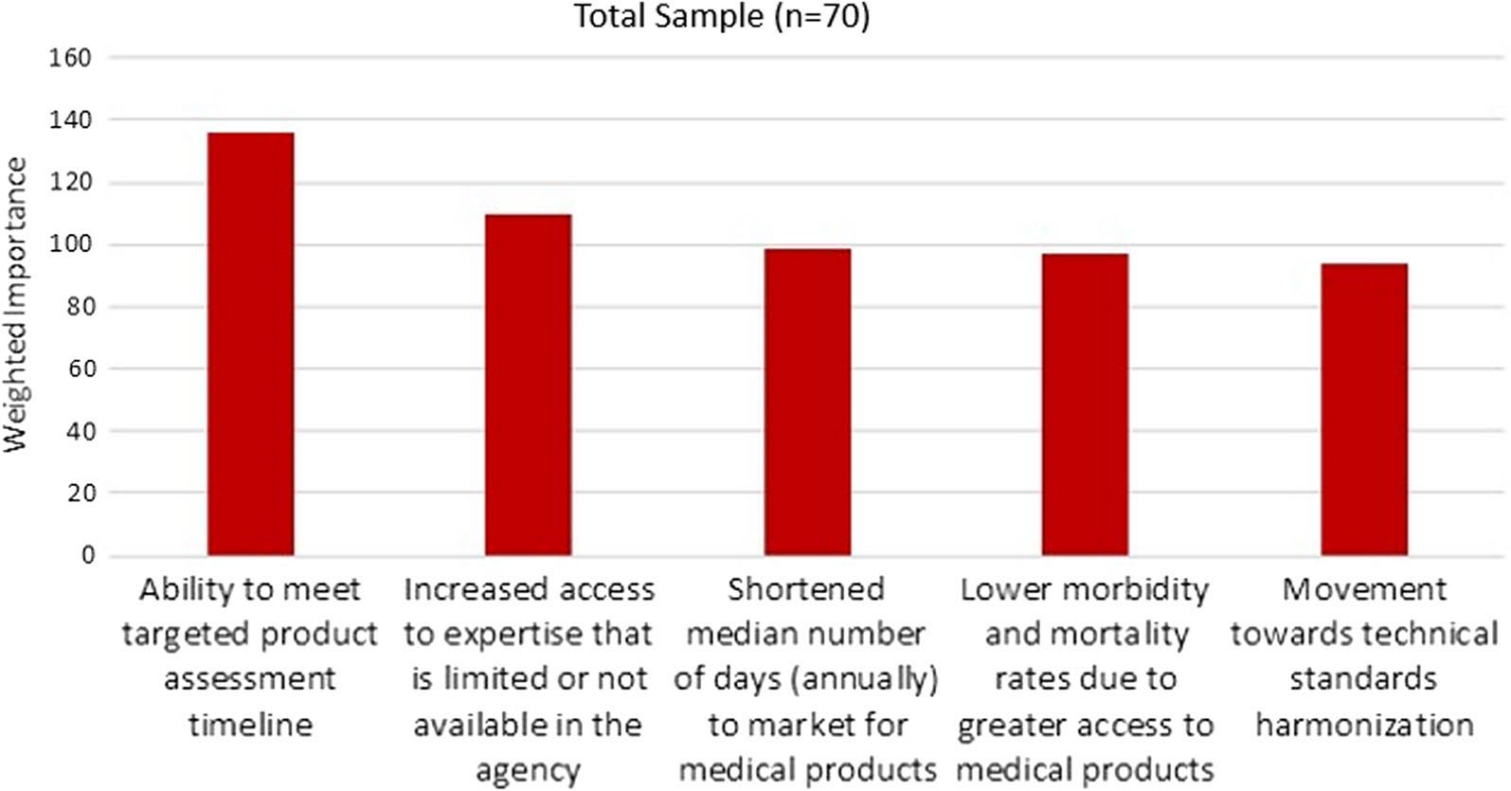
Top Five Metrics Selected by Survey Respondents.

The respondents were asked to choose the Top Five most important metrics to be included in a framework measuring the impacts of a reliance-based agreement, selecting from the metrics listed in Box 2 or any metric they provided. The Top Five results shown in Fig. 1 follow: the ability of an agency to meet targeted product assessment timeline; increased access to expertise that is limited or not available in the agency; shortened median number of days to market for medical products; lower morbidity and mortality due to greater access to medical products; and movement toward technical standards harmonization. The disaggregation of survey data by subgroups (e.g., NGOs, academia, industry) resulted in small subsamples that may be useful for generating hypotheses, but will not be presented in this manuscript.

**Figure 2:**
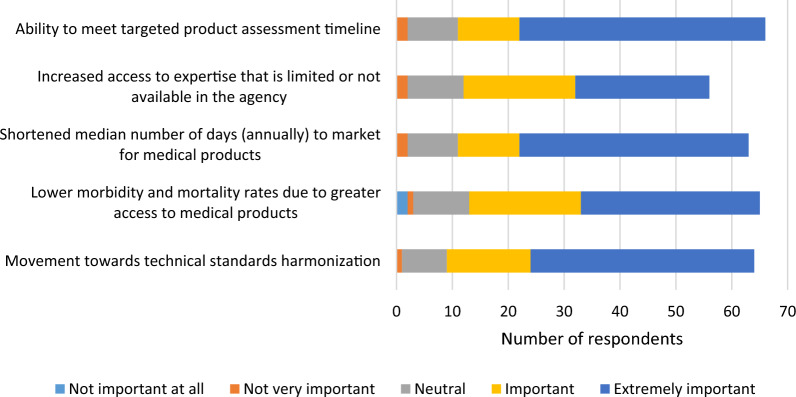
Survey Respondents’ Ranking of the Importance of Including each of the Top Five Metrics in a Framework.

Prior to identifying the Top Five metrics to be included in a framework, respondents were asked to rate the importance of including each metric in a framework that would measure the public health impact or effectiveness of a reliance agreement on a 5-point Likert scale, ranging from “not at all important” to “extremely important.” Below are the results for how they rated the importance of the Top Five metrics (see Fig. 2). Although some participants rated the Top Five metrics as being “not very important” or “not important at all,” taken together, the Likert scale ratings support the “Top Five” results, as the majority of respondents rated each of these metrics as being “important” or “extremely important.” A few respondents skipped some of the Likert scale questions, but still ranked the metrics in the Top Five exercise. It is unclear why this occurred; it is possible that some respondents felt as if they lacked the knowledge to fairly rate all of the Likert scale questions.

### Qualitative Individual In-Depth Interviews (IDIs)

#### Sample

Ten individuals participated in IDIs. Of the 10 IDI participants, five represented regulatory authorities, two of which were from low- or middle-income regions (see Box 3). Other sectors represented included industry (i.e., drug company and generic drugs membership organization), patient advocacy, and international public health.

**Box 3:** IDI Participants’ Locations and Perspective Areas

Regulatory authorities
South American
European
European
North American
Africa
Public health agencies
Latin America/Caribbean
Global Public Health Agency
Patient advocacy
International Patients Advocacy Group
Industry
European Industry/Drug Company
North American Industry/Generic Drug Companies’ Representative

IDIs focused on participants’ opinions about the Top Five metrics as determined by the quantitative survey results. Respondents were not shown the list of novel metrics provided by individual respondents (See Supplementary Material “Other metrics” to be Included in a Framework to Measure the Public Health Impact of a Reliance Agreement (Open-ended responses)). Qualitative results are summarized below.

#### Top Five

##### Ability to Meet the Targeted Product Assessment Timeline and Shortened Median Number of Days to Market for Medical Products

Results for “ability to meet the targeted product assessment timeline” and “shortened median number of days to market for medical products” are presented together, as comments about these metrics overlapped. The IDI participants generally agreed that it would be important to include these two metrics in a framework; however, there were questions as to the usefulness of such indicators for both well-resourced RAs and less well-resourced RAs without additional clarification. For example, shortening a timeline tied to fee regulations was viewed as less meaningful than shortening a timeline that would improve drug lag (i.e., any delay in getting medicines to patients [7]). Others described a need for secondary indicators to understand the local context. Secondary indicators could provide valuable insights into why a product is not being marketed after approval and whether donor programs, not national regulators, are the reasons for delays in patient access.

A sentiment expressed by one RA representative was that every RA considering or engaging in reliance is looking at or collecting data to monitor timelines. Other participants similarly indicated that such data are currently being collected or would be feasible to collect except in instances of RAs with extremely limited resources. Participants representing well-resourced RAs acknowledged that initially, reliance pathways may not save time because protocols must be established; however, they viewed reliance as a long-term commitment, one for which best practices are still being developed. Consequently, these metrics will become more meaningful over time.

##### Increased Access to Expertise that is Limited or Not Available in the Agency

Participants’ perceptions of the benefits of increased access to expertise varied depending on the resource level of the RA. Well-resourced RAs viewed increased access to expertise as a mechanism for collegial scientific discourse but explained that this sort of access does not determine whether a product will be assessed and subsequently approved. Reliance within GMP was briefly mentioned by one well-resourced RA; however, details were not explored, as the focus of the study was on the approval process. Staff from well-resourced RAs often participate in research “clusters” where products are discussed before the respective approval. Participants categorized these collaborations as “peer exchanges” that do not qualify as reliance or even as work-sharing. Two RA representatives expressed organizational reluctance to embrace reliance pathways; one due to a belief among RA staff that they would not have access to the full dossier, and another raised the need for legal provisions in order to use reliance-based pathways.

Participants’ perceptions were that for less well-resourced RAs, increased access to expertise may have a significant impact on assessment. They explained that access to expertise is one of the main reasons less well-resourced RAs use reliance, indicating that this metric is important for less well-resourced RAs taking a more unilateral approach to reliance. With advanced technology and complicated products, predicting future expertise requirements is difficult, especially within less well-resourced RAs. Instead of trying to build capacity within an RA, it would make more sense to engage experts from other RAs through reliance pathways—a way of gaining needed expertise without publicizing a lack of experts. While participants supported the use of this metric, concerns were raised regarding how the metric would be measured and defined. One public health representative suggested assessing the actual demand for advanced technology product applications in lower-resourced environments bringing into question the pressing need for external expertise.

##### Lower Morbidity and Mortality Due to Greater Access to Medical Products

All five participants from RAs felt that lowering morbidity and mortality are noble goals and ethically important, but they did not consider lower morbidity and mortality (LMM) to be an appropriate metric for a reliance framework. Instead, they viewed it as an “impact indicator,” meaning that while LMM might be an outcome of a successful reliance partnership, it is not an RA’s goal. It was consistently stated that regulatory assessments determine the quality, safety, and efficacy of medical products. Furthermore, there are numerous factors that influence the availability of medical products to a regional population. There are many additional steps after regulatory authorization before patients have access to products. RAs are not responsible for what happens after approval; consequently, the majority of participants believed that LMM should not be a Top Five metric for a reliance framework nor should it be a central element, as there are too many other non-regulatory factors that impact a metric such as LMM. The two participants with patient and public health foci were more supportive of including LMM as part of the framework.

Participants questioned how LMM would be measured. In situations where a population is dealing with a specific disease on an epidemic or pandemic scale and therapeutic solutions are limited, it may be possible to measure, to some degree, the impact of approving a product and subsequently making it available in the market, but this is not the norm. RA and industry participants believed that LMM might be a more relevant metric for less well-resourced regions, but still thought there would be challenges to capturing the data, as post-market surveillance of the kinds of data needed to determine LMM in many countries is inadequate.

##### Movement Toward Technical Standards Harmonization

Participants representing RAs of all resource levels directly addressed the issue of alignment and harmonization. The view of one RA representative was that having harmonized standards would facilitate reliance because movement toward technical standards harmonization would set clear guidelines for the critical elements of an inspection. Defining an element through an agreed-upon numerical definition could, the representative suggested, take the ambiguity out of how different RAs interpret the information, thereby minimizing the risk of misunderstandings. Another RA representative agreed saying, “Harmonizing the technical standards would help reliance because it would give me, as Country A, assurance that you in Country B are going to look at and consider what I would have looked at and considered.” To this end, it was suggested that a more important metric may be to understand how each RA interprets and implements the standards. Participants commented that organizations such as ICH, WHO, and PIC/S have already developed technical guidelines for harmonizing to international standards. Much of this effort is being driven by higher-resourced RAs.

#### Beyond the Top Five

##### Missing Metrics

After IDI participants were asked to share their opinions regarding the Top Five, they were asked if there were any metrics that they thought should be added to the framework. The most common suggestion was “access.” The industry representatives strongly supported this metric while two of the RA representatives sought more in-depth information, for example, about drug quality and facilitators or barriers outside of the regulatory process that could affect patient access to medicines. Participants made the point that increasing medicine availability by providing approval for medical products that meet quality, safety, and efficacy standards is the main objective for all RAs; the facilitation of this process is the primary driver behind reliance. However, it is recognized that for this to happen, companies must submit the same product and same information in a timely manner to collaborating RAs. This is something over which regulators have no control. Ensuring patient-level access to these approved medicines is beyond the remit of the regulator, but respondents felt it played a significant role in assessing the impact of a reliance framework.

##### Should There Be One Framework or Multiple Frameworks?

During the interviews, participants often stated that some of the metrics identified in the initial survey were more relevant to less well-resourced RAs or less relevant to well-resourced RAs. Participants who primarily worked with or for well-resourced RAs suggested that most of the metrics were less relevant to their national regulatory practices because these RAs rarely rely on others or rely on others in a more mutual rather than unilateral way. As mentioned elsewhere in this summary and as supported by the quantitative survey data, the importance of LMM as a metric is rated higher for less well-resourced regions. Nevertheless, participants noted that there are still benefits to reliance even for “well-resourced” RAs. Some of the participants suggested that multiple frameworks may be more appropriate. For example, there could be one framework for well-resourced and one for less-well-resourced RAs.

All Top Five metrics were viewed as more important for a framework measuring the public health impact of reliance in less well-resourced RAs. This is due in part to the divide between access to resources for less well-resourced RAs versus well-resourced RAs, which made the selection of metrics difficult and global comparisons inappropriate. A few participants suggested that rather than having a universal framework, regional frameworks and metrics might be more appropriate. One respondent suggested that rather than having a global framework, it may be more useful, especially among less well-resourced RAs to use selected metrics to make comparisons among peers. For example, how does drug lag compare across the region (e.g., neighboring countries) and how has reliance helped to address this? Participants presented one last issue to consider when thinking about the utility of multiple frameworks: the ability of a region or RA to monitor and capture any data necessary for the framework. In some regions, it may be more feasible to focus on collecting only basic data (e.g., “How long did it take for a drug to be assessed?”).

## Discussion

During the interviews, two main themes surfaced. First, well-resourced RAs are unlikely to rely on the assessments of others for the authorization for medicinal products, as they view their access to expertise to be adequate for meeting their current demands. Nevertheless, access to additional expertise may be beneficial for good manufacturing practices (GMP) inspections. Second, while harmonization of technical standards is important, it is more important to understand how each RA interprets and implements the standards. Participants stated that they still want to know how experts from other RAs do their work. They want to know how a product is assessed and how RAs make decisions. It is not enough to have technical standards while simultaneously concealing the evidence or processes that led to the decisions made using those standards. Open discussions allow RAs to develop best practices, so it is not enough to have guidelines for harmonization; RAs globally must understand how to effectively implement the guidelines.

**Figure 3:**
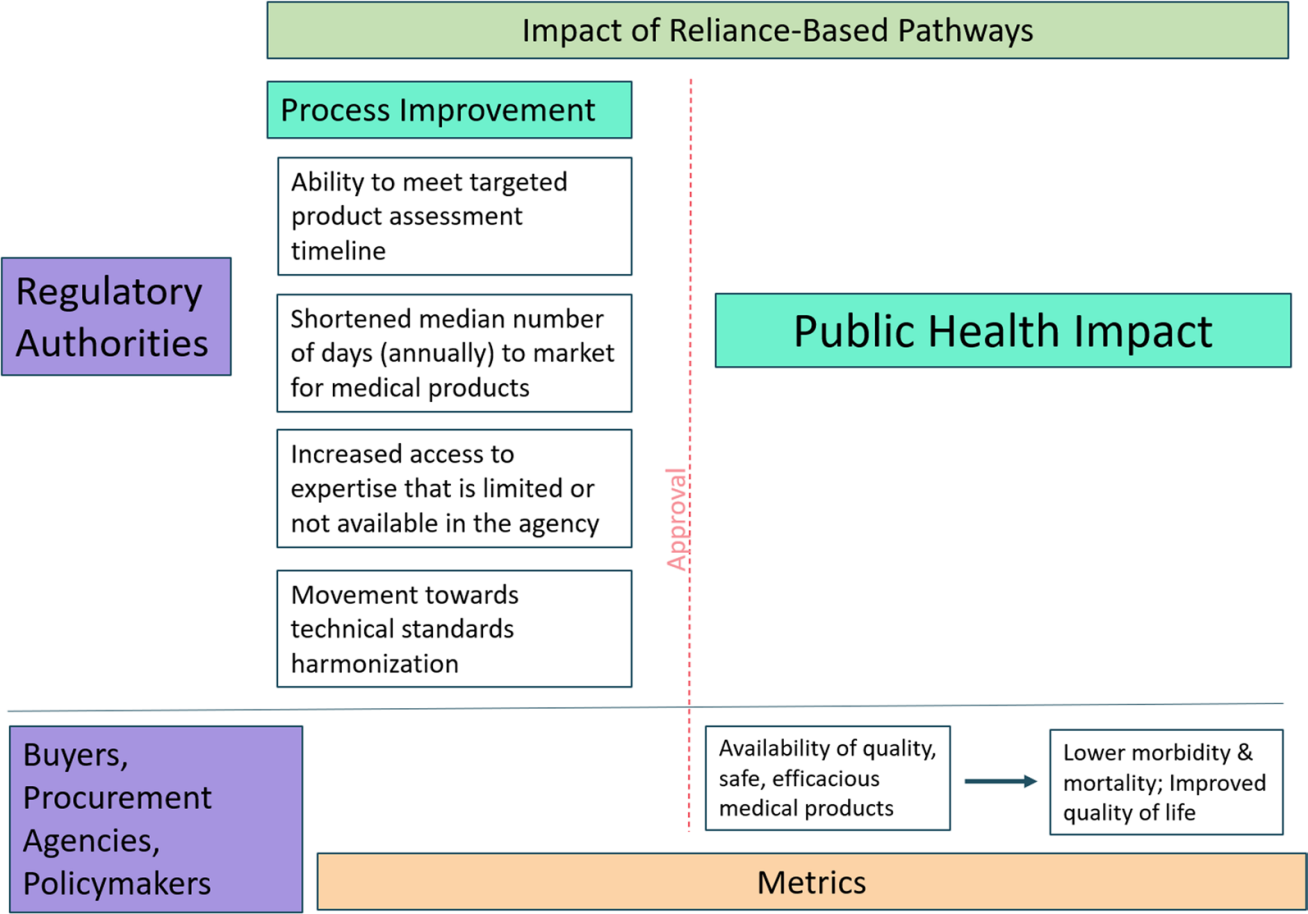
Public Health Impacts of Reliance-Based Pathways.

Based on this small sample, regulators appear to be keenly focused on process—meeting timelines and days to market (see Fig. 3). Other measurements, such as access to medicines, decreased work duplication, improved quality of assessments, and movement toward technical standards harmonization, could be captured to demonstrate the value of reliance to regulators but these metrics stop short of the highest return on investment, building a reliance culture. A reliance culture is founded on trust. Trusted relationships are built over time through collaboration, communication, transparency, and capacity building, with an emphasis on respect for national sovereignty. It is these aspirational metrics that we believe can shift regulatory reliance from process measurements to public health impacts.

These results should be reviewed within the context of a few limitations. We consider this mixed methods study to be a hypothesis-generating study; as such, the quantitative and qualitative samples are small, particularly when one considers the various affiliations represented. Nevertheless, the variety of the sample as well as the agreement among participants both across the phases of the study and among subgroups within each phase are strengths of the study. Second, as survey respondents and IDI participants were invited to participate in this study and volunteered their time, it is possible that individuals who are more passionate or opinionated about reliance-based pathways chose to participate and may not be representative of others involved in reliance-based collaborations; however, the IDI participants in this study are recognized as experts on reliance-based pathways and during the interviews, they were asked to consider the views of their collaborators and colleagues when sharing their opinions. Despite these limitations, this examination of metrics is a valuable addition to the body of research on reliance-based pathways and is useful for informing a framework that can be used to assess the various impacts, including public health impacts, of reliance-based regulatory partnerships.

## Conclusion

In conclusion, interviewed RAs and other stakeholders intuitively believe reliance-based regulatory pathways are a worthwhile endeavor. There must however be “harmonization” within the reliance ecosystem that creates a strong understanding of the factors necessary for reliance-based pathways to be utilized in a successful manner: (a) timely submissions of the same product and the same information by manufacturers to agencies, (b) sharing of complete, helpful documents between agencies in a timely manner, (c) common understandings of how international guidelines are interpreted and implemented by various agencies, and (d) trust among all players in this ecosystem that the system, when it works well, is a win–win for all stakeholders. Without effective, timely collaboration and communication, there will always be excuses for why reliance will not work.

Justifying the value of regulatory reliance to policymakers may be one way of diminishing such resistance to regulatory reliance. For example, focusing on improvements in the efficiency of process elements and concentrating efforts on better use of available human resources could highlight the value. However, linking this increased efficiency and better use of human resources to better public health outcomes is still quite challenging but is necessary to garner policymaker support for expanding reliance activities. This broader view goes beyond the purview of RAs alone and requires greater input from patients, policymakers, and non-governmental and public health agencies to bridge the gap between regulatory assessment decisions and public health impacts.

Future studies exploring metrics for a results framework for public health impacts of regulatory reliance will need to expand the interview base for considering how a “regulatory reliance culture” and the resultant more efficient regulatory system impacts public health. In this way, reliance-based pathways can become valuable regulatory tools for nations wishing to improve their public health.

### Supplementary Information

Below is the link to the electronic supplementary material.

Supplementary file1 (DOCX 25 kb)
