# Supplementary material for: A Mixed Methods Study to Explore Relevant Metrics for a Results Framework Measuring the Public Health Impact of Reliance-Based Pathways
Source: Ther Innov Regul Sci. 2023 Aug 8;57(6):1260–8. doi: 10.1007/s43441-023-00559-5 (PMC10579112; doi:10.1007/s43441-023-00559-5)
Supplement: Supplementary file 1 — Supplementary file1 (DOCX 25 kb) [file 43441_2023_559_MOESM1_ESM.docx]

**SUPPLEMENTARY MATERIAL**

**“Other metrics” to be Included in a Framework to Measure the Public Health Impact of a Reliance Agreement (Open-ended responses).**

*Other metrics to be included:*

1. Reliance activities
2. Resources freed to perform other priority healthcare activities
3. Increase access to complex molecules and vaccines
4. Increased local investment in pharmaceutical sector (e.g., vaccine production) arising from increased maturity of the RA
5. Number of approved NCEs and generics per year
6. Increased capacity with access to assessment reports from other RAs
7. The maturity and stringency of the authority on which the RA is relying
8. Degree of regulatory alignment of assessment outcomes
9. Access to top 20 products known to reduce morbidity and mortality per country
10. Do the reliance processes work well?
11. Technical collaborations of shareholders and RA partners to decide on: 1) how metrics can be standardized; 2) which metrics should be standardized; 3) per country avg. annual income as a regionally standardized metric when purchasing life-saving Rxs such as insulin, immunotherapeutic RXs/therapies, and VBD preventatives.
12. Methods conformity
13. Days used per assessment
14. Independent ethics review; independent safety monitoring; political independence and protection against reprisals
15. Sustainable access to affordable medicines, i.e., post approval variations should also benefit from reliance
16. Cross regulatory reliance—pandemic demonstrated this urgent need
17. Data transparency
18. Post approval change median, number of days to outcome
19. Reduction of questions
20. Expanding # Industry engaging in reliance procedures
21. RWD/RWE for regulatory decisions
22. Agreements on decisions
23. Post-market measures (e.g., tracking safety and effectiveness once approved for marketing)
24. Evidence based decision-making
25. Number of products approved through reliance mechanisms added to national medicines armamentarium
26. Number of products registered through reliance mechanism
27. Shorter times for the access of innovative products
28. Number of approved products from ICH countries
29. Number of market application vs therapeutic class
30. Timelines applicable to essential drugs
31. Legislative or policy support
32. Scope of reliance large (i.e., MAA, LCM, QC testing, inspections, lot release, …) or restricted
33. Recognition of reference products used by generics
34. Improvement managing post-approval changes
35. Packaging harmonization
36. Disease burden
37. Increased cost savings by consumers/insurance fund due to availability of many quality-assured options and increased trust in RA
38. Approval time after first international approval
39. Do the metrics increase scope, depth and quality of the assessment?
40. Test protocols’ conformity
41. Trustworthiness; public accountability
42. Clinical efficacy of medical products
43. Number of outcomes utilizing unredacted and/or redacted assessment reports
44. Number of regulators producing public assessment reports that are used for reliance
45. Data for efficient decision-making in health
46. Periodic comparisons of outcomes (e.g., which products approved or denied in each jurisdiction; reasons; extent of reliance)
47. Training
48. More access, lower prices
49. Monitor use and results from use in a particular disease condition (e.g., chronic foot ulcers or amputations in people with diabetes)
50. Similar global submission or approval timelines
51. Bioequivalence studies regulation across Latin America

*Responses that reiterated the original 11 metrics:*

1. Cost savings
2. Standards harmonization
3. Movement towards harmonization of regulatory procedures (not the technical requirements, but the way the requirements are processed by agencies)
4. Decreased population morbidity and mortality
5. Ability to meet targeted product assessment timeline
